# Metabolomic Profile of Umbilical Cord Blood Plasma from Early and Late Intrauterine Growth Restricted (IUGR) Neonates with and without Signs of Brain Vasodilation 

**DOI:** 10.1371/journal.pone.0080121

**Published:** 2013-12-02

**Authors:** Magdalena Sanz-Cortés, Rodrigo J. Carbajo, Fatima Crispi, Francesc Figueras, Antonio Pineda-Lucena, Eduard Gratacós

**Affiliations:** 1 Department of Maternal-Fetal Medicine, Institute Clinic of Gynecology, Obstetrics and Neonatology, Hospital Clinic, Institut de Investigacions Biomèques August Pi-Sunyer (IDIBAPS), Universitat de Barcelona, Barcelona, Spain; 2 Structural Biochemistry Laboratory, Centro de Investigación Príncipe Felipe, Valencia, Spain; Hôpital Robert Debré, France

## Abstract

**Objectives:**

To characterize via NMR spectroscopy the full spectrum of metabolic changes in umbilical vein blood plasma of newborns diagnosed with different clinical forms of intrauterine growth restriction (IUGR).

**Methods:**

23 early IUGR cases and matched 23 adequate-for-gestational-age (AGA) controls and 56 late IUGR cases with 56 matched AGAs were included in this study. Early IUGR was defined as a birth weight <10^th^ centile, abnormal umbilical artery (UA) Doppler and delivery <35 weeks. Late IUGR was defined as a birth weight <10^th^ centile with normal UA Doppler and delivery >35 weeks. This group was subdivided in 18 vasodilated (VD) and 38 non-VD late IUGR fetuses. All AGA patients had a birth weight >10^th^ centile. ^1^H nuclear magnetic resonance (NMR) metabolomics of the blood samples collected from the umbilical vein at delivery was obtained. Multivariate statistical analysis identified several metabolites that allowed the discrimination between the different IUGR subgroups, and their comparative levels were quantified from the NMR data.

**Results:**

The NMR-based analysis showed increased unsaturated lipids and VLDL levels in both early and late IUGR samples, decreased glucose and increased acetone levels in early IUGR. Non-significant trends for decreased glucose and increased acetone levels were present in late IUGR, which followed a severity gradient when the VD and non-VD subgroups were considered. Regarding amino acids and derivatives, early IUGR showed significantly increased glutamine and creatine levels, whereas the amounts of phenylalanine and tyrosine were decreased in early and late-VD IUGR samples. Valine and leucine were decreased in late IUGR samples. Choline levels were decreased in all clinical subforms of IUGR.

**Conclusions:**

IUGR is not associated with a unique metabolic profile, but important changes are present in different clinical subsets used in research and clinical practice. These results may help in characterizing comprehensively specific alterations underlying different IUGR subsets.

## Introduction

Intrauterine growth restriction (IUGR) affects 7-10% of all pregnancies [1] and is defined by the underachievement of the genetic growth potential in the fetus. IUGR is associated with an increased risk for adverse perinatal outcome [2–4] and long term fetal programming in the form of cardiovascular disease, metabolic syndrome and neurological deficits [5–8]. The early-onset forms of IUGR represent the most severe [6,9] but less prevalent presentation of this condition. Early-onset IUGR is consistently associated with abnormalities in feto-placental Doppler and with severe placental insufficiency [10–12]. Late-onset forms of IUGR, -also referred to as small for gestational age (SGA) [2]-, are far more prevalent than early IUGR, but they represent a more heterogeneous condition. While, as a whole, late-onset IUGR is associated with signs of placental injury and poorer perinatal outcome [13], there are important individual differences in the feto-placental Doppler response and a proportion of these fetuses present with relatively normal perinatal outcomes. There is general agreement that it is likely that different causes may lead to late-onset IUGR [14] and that part of late IUGR fetuses are merely constitutionally small [15]. However, clinical clues to differentiate specific groups within this diagnostic category are still scarce. Research over recent years has demonstrated that a subgroup of late-onset IUGR have signs of increased brain perfusion as measured by middle cerebral artery (MCA) Doppler [16].This subset has consistently been reported to present poorer perinatal outcome [16–19]. In contrast, fetuses with normal brain Doppler have similar outcomes when compared to fetuses with normal growth [16,19]. Since brain vasodilation is a response to hypoxia [20], it has been suggested that late IUGR fetuses with increased brain perfusion represent milder forms of a “late-onset placental disease” group with milder but similar features to early-IUGR fetuses [16,21]. However, there are no grounds to support these assumptions. In addition, it is unknown whether those SGA fetuses with no Doppler changes in brain circulation are merely milder forms of the same disease, whether they are constitutionally small fetuses, or whether they represent another pathogenic pathway leading to abnormally low fetal growth.

Metabolomics is considered a powerful approach to study the multivariate metabolic responses to physiological and/or pathological stressors, providing integrative information about patterns of disease [22–24]. Recently, two studies reported that the metabolic blood profiles of IUGR newborns express significant differences in glucose and amino acid metabolic levels in comparison with controls [25,26]. However, the specific metabolomic patterns of the different clinical forms of IUGR have not been investigated. We hypothesized that metabolomics could be helpful in elucidating whether there are pathophysiological differences behind the above described IUGR subsets.

The aim of this prospective study was to characterize the full spectrum of metabolic changes in cord blood plasma of newborns diagnosed with different clinical forms of IUGR. We used a metabolomics approach based on ^1^H-NMR spectroscopy. Three clinical groups of early-onset IUGR, late-onset IUGR with normal brain Doppler and late-onset IUGR with Doppler signs of vasodilation were recruited and compared with matched normally grown neonates. 

## Materials and Methods

This study was conducted at the Maternofetal Medicine Research Group, Hospital Clinic, Barcelona Spain and at the Centro de Investigación Príncipe Felipe, Valencia, Spain. The study protocol was approved by the Hospital Clinic of Barcelona ethics committee and all patients that were included for this study provided a written informed consent for their inclusion in the study and the blood obtained from their neonates as their legal guardians. IRB (2012/7684)

### Study populations

The study design was a prospective cohort study that included 79 cases diagnosed with IUGR (23 early and 56 late IUGR) and 79 control subjects (23 matched for early IUGR and 56 for late IUGR) with a birth weight appropriate for gestational age. Pregnancies were dated by the first-trimester crown-rump length measurement [27]. Patients that were enrolled in this study were identified before delivery from singleton pregnancies by performing a detailed ultrasound-Doppler assessment within one week before delivery. In all study participants full prenatal information was available. Our sample was classified in early and late IUGR defined as an estimated and confirmed birth weight below the 10^th^ centile according to local standards [28]. Additional defining criteria for early IUGR fetuses: 1) Gestational age at birth 34 weeks or less; 2) Umbilical artery Doppler pulsatility index (PI) >95^th^ centile [29] in at least two consecutive examinations 24 hours apart. Late IUGR neonates were also defined by: 1) Gestational age at delivery equal or above 35 weeks; 2) Normal umbilical artery Doppler PI (below 95^th^ centile) [29]. Late IUGR samples were divided into two subgroups: Vasodilated late-IUGR if they presented a MCA PI value <5^th^ centile [30] in at least two consecutive examinations 24 hours apart and non-vasodilated late IUGR cases if their MCA PI was >5^th^ centile. Control subjects were singleton appropriate-for-gestational-age (AGA) fetuses (birth weight >10^th^ centile according to local standards [28]) that were matched with cases by gestational age at delivery (± 7 days) and gender. Subjects were considered non-eligible in the presence of any of the following: Congenital malformations or chromosomal defects, congenital infections, clinical chorioamnionitis or maternal disease such as autoimmune disease or the presence of cardiovascular risk factors such as diabetes mellitus, pregestational dyslipidemia or BMI >35.

### Clinical and Ultrasound parameters

All IUGR fetuses underwent a detailed anatomical and Doppler examination. Prenatal Doppler parameters such as umbilical artery PI and MCA PI were prospectively recorded. Prenatal Doppler ultrasound examinations were performed using a Siemens Sonoline Antares scanner (Siemens medical systems, Malvern, PA, USA) and a 6-2 MHz linear-curved-array transducer. Spectral Doppler parameters were obtained automatically from three or more consecutive waveforms with the angle of insonation as close to zero as possible. Umbilical artery PI was obtained on a free floating cord loop. MCA PI was obtained in a transversal view of the fetal head, at its origin from the circle of Willis. 

The administration of oral or endovenous maternal drugs two weeks before or at delivery was recorded in all IUGR cases and controls.

### Samples

500 µl of blood was obtained from the umbilical vein after cord clamping at delivery from each subject and kept in EDTA-treated tubes. All samples were processed within 1 hour. Plasma was separated by centrifugation at 3000 rpm for 10 minutes at 4°C; samples were stored immediately at -80°C in aliquots such that freeze-thawing was minimized. Samples were transported in dry ice for the metabolomics analysis. 

Preparation of neonatal serum samples for metabolomic analysis by ^1^H-NMR was carried out according to established protocols [31,32]. Before analysis, the samples were thawed at room temperature. 200 µl of sample was mixed with 400 µl of 0.9% saline solution (wt/vol, NaCl in 90% H_2_O/10% D_2_O) and then centrifuged at 11,400 rpm for 5 min at 4°C. 550 µl of the supernatant was added to a 5 mm NMR tube, kept in ice, and left at room temperature for 30 minutes before measurement. Case and control samples were selected for analysis in a random order. 

A preparation of the drugs that were administered to the mother before or at delivery was obtained for NMR analysis (100% D_2_O, 500 µl final volume). 

### NMR Spectroscopy

All spectra were recorded at 27°C with a Bruker Ultrashield Plus Avance II 600 MHz spectrometer (Bruker Biospin, Rheinstetten, Germany) equipped with a 5-mm cryogenically-cooled TCI probe. For each serum sample Carr-Purcell-Meiboom-Gill (CPMG) [33] and noesy1D spectra were acquired. The CPMG relaxation-editing pulse sequence was collected with a total of 128 accumulations and 32K data points over a spectral width of 20 ppm. The number of loops was set to total 40 ms of spin-spin relaxation delay and a 3 s relaxation delay was included between FIDs during which water irradiation was applied to suppress the H_2_O signal. ^1^H noesy1D spectra were collected using 128 scans, 32K data points, 20 ppm of spectral width, 4 s relaxation delay and 10 ms of mixing time. Water presaturation was applied during both relaxation and mixing times. For both experiments a line broadening of 1.0 Hz was applied to the FID which was zero-filled to double the number of Fourier domain points. Spectra were phased manually and the baseline corrected automatically using TopSpin (v. 1.3, Bruker BioSpin, Rheinstetten, Germany). Chemical shifts were referenced internally to the CH_3_ resonance of alanine at 1.47 ppm. To aid in metabolite identification 2D *J*-resolved (8 transients and 80 increments), ^1^H,^1^H-TOCSY and ^13^C-HSQC experiments were acquired for selected samples, using 256-512 increments, 32-128 transients and 1.5 s for relaxation delay. For reference and possible interferences in metabolite identification, ^1^H NMR spectra in 100% D_2_O (32 scans, 32K data points, 14 ppm of spectral width) were recorded for all the maternal drugs supplied. 

### Data processing and statistical analysis

Each NMR spectrum was used to construct data matrices for the statistical analysis by dividing it (from 10.0 to 0.0 ppm) into buckets of fixed (0.05 ppm) or variable sizes that were integrated using Amix (v. 3.9.7, Bruker BioSpin, Rheinstetten, Germany). The water resonance region (5.15-4.40 ppm) was excluded from the analysis. Buckets were normalized to the sum of total spectral intensity to minimize potential differences in concentration between samples. The identification of metabolites was performed through a combination of ^1^H/^13^C chemical shifts, *J*-couplings, TOCSY NMR data and their comparison to metabolite NMR parameters described in literature references 34,35. For the quantification of selected metabolites, the region or regions corresponding to their NMR signals in the CPMG spectra were integrated. The areas in between the relevant regions were also integrated to fully cover the spectral width of the ^1^H (0-10 ppm) with the exception of the water signal region (4.40-5.15 ppm) that was conveniently excluded. The integrated spectrum was then normalized to total area. Results were presented as mean ± standard error of the mean (s.e.m.). Statistical significance of the data was determined using the non-parametric Mann-Whitney U test or ANOVA (Tukey-Kramer post hoc test) where appropriate. P-values < 0.05 were considered to be statistically significant. 

Multivariate data analysis was performed using SIMCA-P+ v. 12.0 (Umetrics AB, Umeå, Sweden). Data were scaled using the Pareto method (each value divided by the square root of the standard deviation of each variable), which is normally the best choice in the case of spectroscopic data as it decreases the relative importance of highly abundant metabolites but at the same time preserves data structure. Mean centering was applied to the scaled data. Unsupervised principal components analysis (PCA) was used for the overview of individual classes and all classes jointly, to observe clustering or separation trends and for the identification of outliers. PCA loading plots were used to identify metabolites responsible for any separation observed. Supervised multivariate analysis was carried out with Partial Least Squares Discriminant Analysis (PLS-DA) and orthogonal PLS-DA (OPLS-DA). Evaluation of the PLS/OPLS-DA models was performed using the goodness-of-fit parameter *R* ^*2*^ *Y* (variation in class membership explained by the model) and the predictive ability parameter *Q* ^2^ (goodness of prediction, calculated by 7-fold internal cross-validation). Models were externally validated by building a model with 2/3 of the data set and predicting the remaining 1/3 in turn, using Fisher’s probability as quality probe of the classification (<0.01 generally considered a good prediction). Classification results were used to define True Positives (TP), True Negatives (TN), False Positives (FP) and False Negatives (FN) and therefore calculate as percent sensitivity [TP/(TP + FN) × 100] and specificity [TN/(TN + FP) × 100]. The CV-ANOVA (Analysis Of Variance testing of Cross-Validated predictive residuals) included in SIMCA was also used for assessing the reliability of the PLS/OPLS models. P-values (likelihood of obtaining such a classification by chance) lower than 0.05 were indicative of a significant model. In those OPLS models showing clear separation between classes in the score plots, the metabolites responsible for the separation were identified from the loading and the predictive S-plots (combination of modelled covariance and modelled correlation in a scatter plot). Those variables presenting high magnitude and reliability in the S-plot and adequate confidence intervals in the loading plot were further investigated for putative biomarker identification. 

Student’s t test for independent samples and Pearson’s X^2^ or Fisher’s exact tests were used to compare quantitative and qualitative clinical and demographic data respectively using SPSS statistical software, version 17.0 (SPSS for Windows, SPSS Inc, Chicago, IL, USA). A *p* value < 0.05 was considered statistically significant.

## Results

### Study populations

From the initially selected samples, 3 obtained from early IUGR cases and 1 AGA sample from the late subset had to be excluded due to the detection of sample contamination at the moment of aliquotation (3) and the repeal from the parents to be included in this study (1), leaving 20 cases vs. 23 controls in the early subset and 56 cases vs. 55 controls in the late subset for metabolomic analysis.

Table 1 shows the clinical characteristics of the early IUGR vs. AGA comparison (early subset). Early IUGR group shows an increased rate of preeclampsia and caesarean section delivery, with similar rates of neonatal acidosis, abnormal Apgar score at 5 minutes and maternal steroid administration. Maternal drug administration in this subset was recorded and shown in Table S1. Administration of MgSO_4_ for the treatment of severe preeclampsia was significantly higher in early IUGR patients. Administration of antibiotics (amoxicillin-clavulanic, penicillin, erythromycin, ampicillin and gentamicin were most commonly used) and tocolytic agents (ritodrine, nifedipine or atosiban as most commonly used) as a consequence of premature rupture of membranes (PROM) and preterm labour were significantly higher in preterm controls.

**Table 1:** Maternal and neonatal clinical characteristics of the study group: Early Subset.

	**Early IUGR**	**AGA**	***P***
	**(N=20)**	**(N=23)**	
Maternal age (y)	31.5 ± 5.6	32.3 ± 3.8	0.56
Maternal BMI (kg/m^2^)	24.1 ± 4.0	22.7 ± 2.7	0.25
Maternal smoking (%)	20.0	34.8	0.28
PROM (%)	5.0	69.6	<.001
Premature labor (%)	20.0	65.2	<.01
Preeclampsia (%)	50	0	<.001
GA at birth	31.7 ± 2.2	31.5 ± 2.4	0.83
Cesarean section (%)	95.0	21.7	<.001
Birth weight (g)	1114.7 ± 383.5	1788.2 ± 445.1	<.001
Weight centile	1.0 ± 2.6	41.0 ± 31.4	<.001
Male/female	9/11	13/10	0.45
Neonatal acidosis* (%)	25	14.3	0.29
Apgar score at 5 minutes <7 (%)	30	8.7	0.07
Lung maturation with steroids (%)	80	69.6	0.43
Days from steroids to delivery	2.69 ± 2.1	2.44 ± 3.3	0.8

Results are expressed as mean ± standard deviation or percentage determined by Student´s t-test for independent samples, Pearson´s X^2^ or Fisher´s exact test as appropriate. y=years; PROM: Premature rupture of membranes; GA: Gestational age; * Neonatal acidosis defined as umbilical artery pH<7.15 and base excess > 12 mEq/l.

Tables 2 to 4 show clinical characteristics of the late IUGR vs. controls comparison (late subset with and without brain vasodilation). Late IUGR shows an increased rate of maternal smoking status at booking, with similar perinatal results to their AGA counterparts. Maternal drug administration in the late subset was also recorded finding significantly higher rates of antibiotic administration in controls as shown in Table S2. 

**Table 2:** Maternal and neonatal clinical characteristics of the study group: Late Subset.

	**Late IUGR**	**AGA**	***P***
	**(N=56)**	**(N=55)**	
Maternal age (y)	32.1 ± 5.1	33.1 ± 5.2	0.34
Maternal BMI (kg/m^2^)	22.7 ± 4.1	24.8 ± 4.6	0.02
Maternal smoking (%)	30.4	10.9	0.01
Preeclampsia (%)	8.9	1.8	0.09
GA at birth	38.3 ± 1.9	38.7 ± 1.8	0.11
Cesarean section (%)	42.9	29.1	0.13
Birth weight (g)	2314.6 ± 286.5	3153 ± 446.3	<0.001
Weight centile	3.8 ± 4.8	48. 2 ± 22.7	<0.001
Male/female	29/27	26/29	0.63
Neonatal acidosis* (%)	9.6	1.9	0.09
Apgar score at 5 minutes <7 (%)	7.1	1.8	0.17

Results are expressed as mean + and standard deviation or percentage determined by Student´s t-test for independent samples, Pearson´s X^2^ or Fisher´s exact test as appropriate. y=years; GA: Gestational age; g= grams; * Neonatal acidosis defined as umbilical artery pH<7.15 and base excess > 12 mEq/l.

**Table 3:** Maternal and Neonatal Clinical Characteristics of the Study group: Late vasodilated IUGR vs. AGA fetuses.

	**Late IUGR**	**AGA**	***P***
	**(N=18)**	**(N=17)**	
Maternal age (y)	33.3 ± 6.3	33.5 ± 3.3	0.91
Maternal BMI (kg/m^2^)	22.2 ± 4.7	24.1 ± 4.7	0.26
Maternal smoking (%)	27.8	5.9	0.08
Preeclampsia (%)	16.7	5.9	0.3
GA at birth	38.4 ± 1.3	38.7 ± 2.0	0.67
Cesarean section (%)	55.6	29.4	0.11
Birth weight (g)	2373.1 ± 400.7	3117.1 ± 500.2	<0.001
Weight centile	5.2 ± 7.2	46.20 ± 23.9	<0.001
Male/female	12/6	10/7	0.63
Neonatal acidosis* (%)	20	0	0.05
Apgar score at 5 minutes <7 (%)	16.7	0	0.07

Results are expressed as mean + and standard deviation or percentage determined by Student´s t-test for independent samples, Pearson´s X^2^ or Fisher´s exact test as appropriate. y=years; GA: Gestational age; UA: Umbilical artery; g= grams; * Neonatal acidosis defined as umbilical artery pH<7.15 and base excess > 12 mEq/l.

**Table 4:** Maternal and Neonatal Clinical Characteristics of the Study group: Late non-vasodilated IUGR vs. AGA fetuses.

	**Late IUGR**	**AGA**	***P***
	**(N=38)**	**(N=38)**	
Maternal age (y)	31.6 ± 4.3	32.9 ± 5.9	0.27
Maternal BMI (kg/m^2^)	22.9 ± 3.8	25.1 ± 4.6	0.04
Maternal smoking (%)	31.6	13.2	0.05
Preeclampsia (%)	5.3	0	0.15
GA at birth	38.3 ± 1.1	38.8 ± 1.7	0.9
Cesarean section (%)	36.8	28.9	0.46
Birth weight (g)	2287 ± 214.1	3169 ± 426.3	<0.001
Weight centile	3.3 ± 3.3	49.7 ± 22.3	<0.001
Male/female	17/21	16/22	0.7
Neonatal acidosis* (%)	5.4	2.9	0.58
Apgar score at 5 minutes <7 (%)	2.6	2.6	1

Results are expressed as mean + and standard deviation or percentage determined by Student´s t-test for independent samples, Pearson´s X^2^ or Fisher’s exact test as appropriate. y=years; GA: Gestational age; UA: Umbilical artery; g= grams; * Neonatal acidosis defined as umbilical artery pH<7.15 and base excess > 12 mEq/l.

### NMR analysis

1D CPMG and noesy1D NMR spectra were recorded for each of the IUGR samples (early, N = 20 and late, N = 56) and their matched controls (N = 23 and 55 respectively).

### Early-onset IUGR vs. matched AGA newborns (early subset)

**Figure 1:**
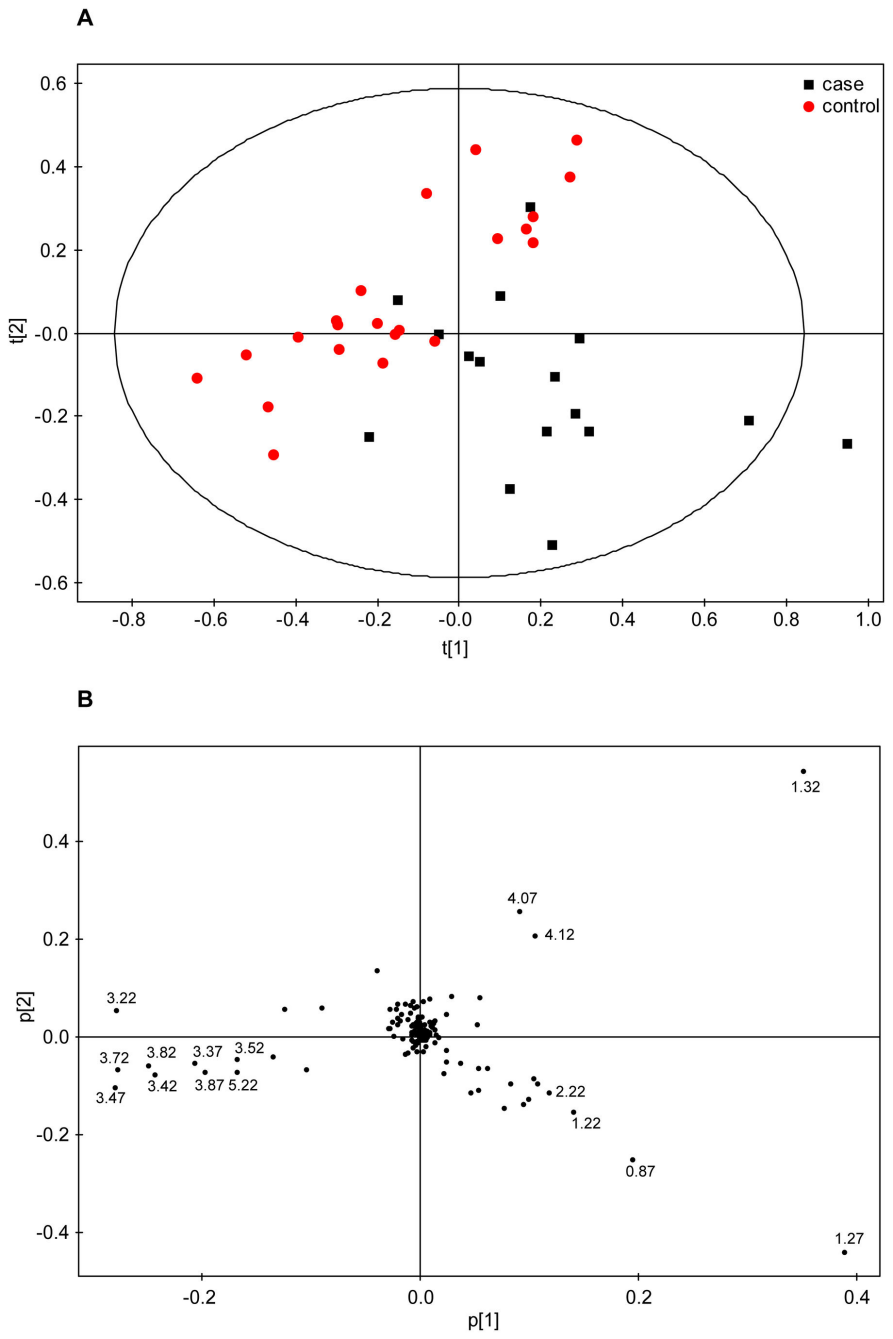
Multivariate statistical analysis of early IUGR. (a) PCA scores-plot of the ^1^H-CPMG spectra from controls (N = 22, red dots) and cases (N = 16, black squares). The first two principal components, PC1 and PC2, explain 42 and 20% of the variation respectively. (b) PCA loadings of the ^1^H-CPMG spectra displaying the correlated structure of the variables. Selected loadings are labelled with the midpoint of the corresponding NMR bucket (in ppm). The buckets responsible for the case/control differentiation are those separating away from the central cluster and in the same direction of the case/control samples observed in the corresponding PCA scores plot in (a).

In order to perform the metabolomic analysis of the early IUGR and matched AGA newborn samples and to spot possible outliers, preliminary unsupervised PCA was applied to the full NMR data (43 samples) as well as separately to the early IUGR cases and AGA control subgroups. Four cases and one control samples were consistently located in the PCA scores plot outside the 0.95 Hotelling’s T2 confidence interval. These five strong outliers required further investigation and their NMR raw data were examined in detail: One of the above case samples showed its NMR spectra dominated by very intense broad signals some of which could be attributed to lipids. In this particular case, the clinical data presented some peculiar features (e.g. lowest weight of the early IUGR subset, 400 gr) that suggested severe neonatal distress. Its exclusion from the following study was therefore justifiable to avoid a potentially biased analysis. In the case of the control sample singled out as outlier, it presented very intense glucose signals that overshadowed the rest of the NMR spectrum, which was compatible with the maternal diagnosis of suboptimally controlled gestational diabetes. This prompted us to exclude it from the study as well. The other three IUGR cases identified as strong outliers presented several common signals that largely dominated the spectra: four singlets at 2.55, 2.69, 3.20, 3.61 ppm and two AB patterns at 3.11 and 3.22 ppm split with the characteristic ^2^ *J*_HH_ spin coupling. These signals were identified as exogenous to the biological samples and assigned to the EDTA (ethylenediaminetetraacetic acid) used as anticoagulant in the collection tubes[36]. Thus, the intense singlets at 3.20 and 3.60 ppm correspond to the NCH_2_CH_2_N and NCH_2_CO protons of free-EDTA, respectively, whereas the pair 2.55/3.11 ppm arises from the Ca-EDTA complex and the lower intensity signals at 2.69/3.22 ppm from the Mg-EDTA one. Several other samples were identified as moderate outliers via the observation residuals detected with the SIMCA-P+ tool DModX (distance to model), but in each case the deviation could be attributed to intense signals from a particular metabolite. Furthermore, no unusual characteristics were associated with the source of these samples or their mothers that could justify their exclusion from the analysis and they were therefore retained for the statistical study. After the removal of the five strong outliers, the PCA scores plot for the early subset data showed a reasonable separation between IUGR cases and controls (*R* ^2^= 0.63; *Q* ^2^= 0.37 for the first two components; Figure 1a): Controls are positioned in the upper left part of an imaginary line that crosses the scores plot diagonally, whereas most early-onset IUGR cases are located in the lower right section. The SIMCA P+ loadings plot (Figure 1b) provided qualitative information about which variables (i.e. NMR buckets) were responsible for the class separation observed in the PCA. For this particular model, the NMR signals that primarily contributed to the clustering of the IUGR cases were mainly located between 0.80-1.35 ppm (appearing on the right half of the loadings plot), an NMR region characteristic of lipids, lactate and the methyl groups from amino acids such as valine, leucine, isoleucine and threonine. The differentiation of the control samples arises from NMR buckets in the region 3.20-4.00 ppm (Figure 1b, left half), which correspond to metabolites like α/β-glucose or the alpha and beta hydrogen atoms of most amino acids. Likewise, a bucket at 5.22 ppm attributable to the H1 of α-glucose, also contributed significantly to the discrimination of the control samples. Therefore, a qualitative interpretation of the loadings plot pointed to glucose as a relevant metabolite in the early IUGR cases/controls differentiation.

The PCA was also applied to examine the influence in the score plot distribution of the maternal and neonatal clinical characteristics of the study group (Table 1). Thus, samples were labelled in a discrete way (0, 1) for variables such as smoking, PROM, premature labour, preeclampsia, caesarean vs. vaginal delivery, gender, neonatal acidosis and abnormal Apgar score. Subgroups were created such that they covered the whole sample range for those characteristics represented by a continuous variable (maternal age, BMI, GA at delivery, birth weight). However, no sample clustering or group differentiation was observed for any of the above clinical characteristics, except for the way of delivery, which showed a similar separation to that observed between cases and controls. This trend is easily explained since over 95% of early IUGR cases were delivered by caesarean section compared to 21.7% of the matched controls. The potential bias caused by the administration of maternal drugs was also considered on the PCA analysis, but no related sample discrimination was observed.

**Figure 2:**
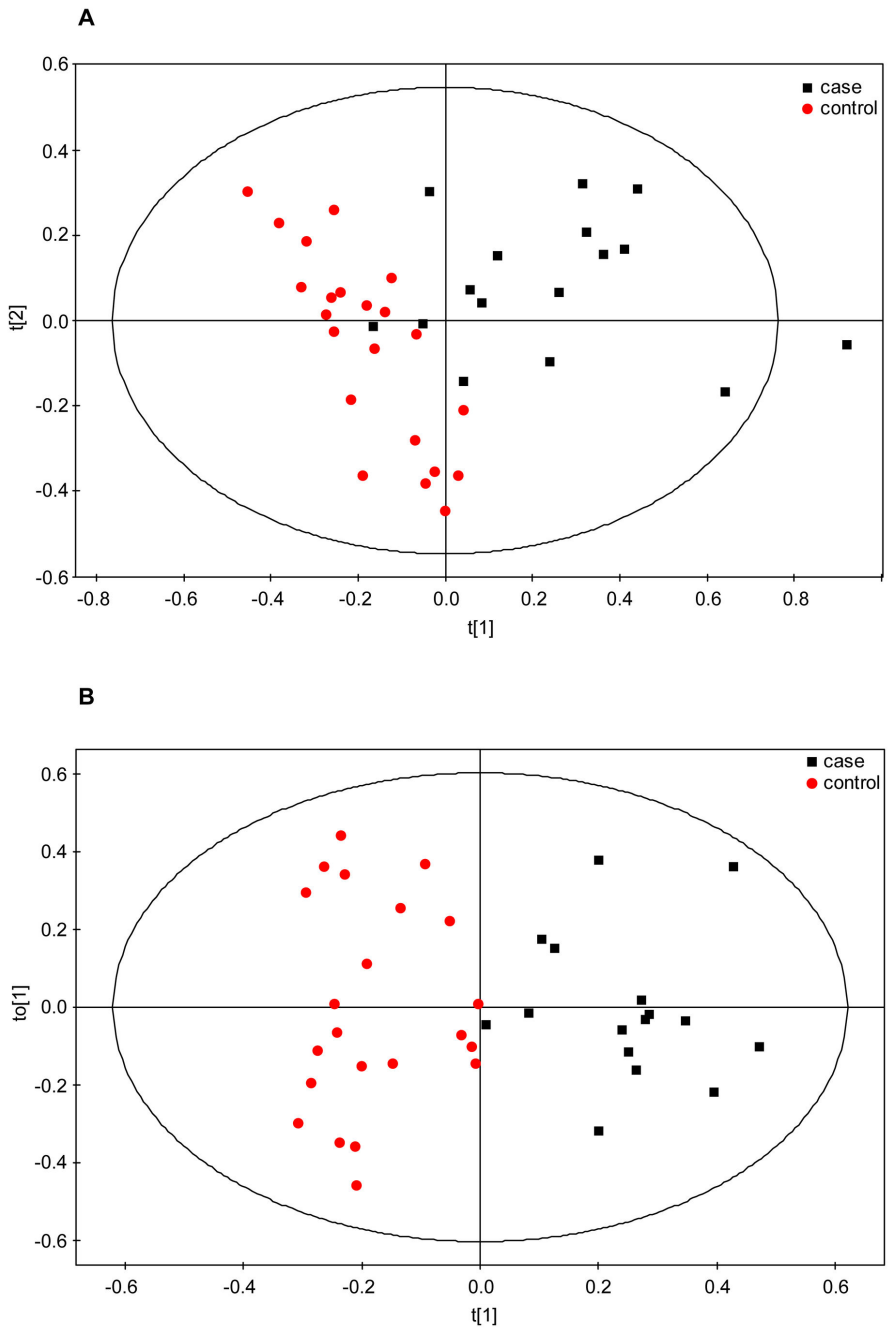
Multivariate statistical analysis of early IUGR. (a) PLS-DA (*R* ^2^ *Y* = 0.64*; Q* ^2^ = 0.54; *p*-value = 2.8E-05) and (b) OPLS-DA (*R* ^2^ *Y* = 0.79; *Q* ^2^ = 0.58; *p*-value = 0.00052) scores plots of the ^1^H-CPMG spectra from controls (N = 22, red dots) and cases (N = 16, black squares). Samples from cases and controls were defined as two different classes.

The NMR data from the early subset was further examined through supervised multivariate PLS-DA, where case/control class membership is defined with the discrete variable Y. This analysis produced a significant separation between the two groups as observed in the corresponding scores plot (Figure 2a). Class separation was moderately improved in the OPLS-DA model (Figure 2b), presenting goodness-of-fit *R* ^*2*^ *Y* = 0.79 and goodness-of-prediction *Q* ^2^ = 0.58 (*Q* ^2^ > 0.5 generally considered a good model in metabolomics) (i.e. the model explains 79% of the variation between cases and controls with a predictive ability of 58%). Several validation tests were applied to the model for quality and prediction measurement (Table 5) that confirmed its robustness. 

**Table 5:** IUGR multivariate statistical analysis parameters and model validation values.

	*Model*	*R^2^X*	*R^2^Y*	*Q2*	*p* ^a^	*Sensitivity**^b^***	*Specificity**^b^***	*Fishers prob.**^c^***
Early	PLS-DA	0.61	0.64	0.54	2.84E-05	100	88	1E-07
Early	OPLS-DA	0.75	0.79	0.58	0.0005	100	95.6	1E-09
Late (all)	PLS-DA	0.45	0.22	0.07	0.133	66	64	0.0018
Late (all)	OPLS-DA	0.71	0.30	0.17	0.005	70.7	78	8.3E-07
Late (VD)	PLS-DA	0.59	0.47	0.32	0.022	77.8	75	0.0027
Late (VD)	OPLS-DA	0.59	0.47	0.32	0.019	77.8	75	0.0027

^a^ *p* values were obtained from a two-way analysis of variance (ANOVA). ^b^ Classification results were used to define True Positives (TP), True Negatives (TN), False Positives (FP) and False Negatives (FN) and therefore calculate as percent sensitivity [TP/(TP + FN) × 100] and specificity [TN/ (TN + FP) × 100]. ^c^ Probability of a particular classification result occurring by chance and is satisfied when p<0.05 for 95% confidence. VD: Vasodilated.

**Figure 3:**
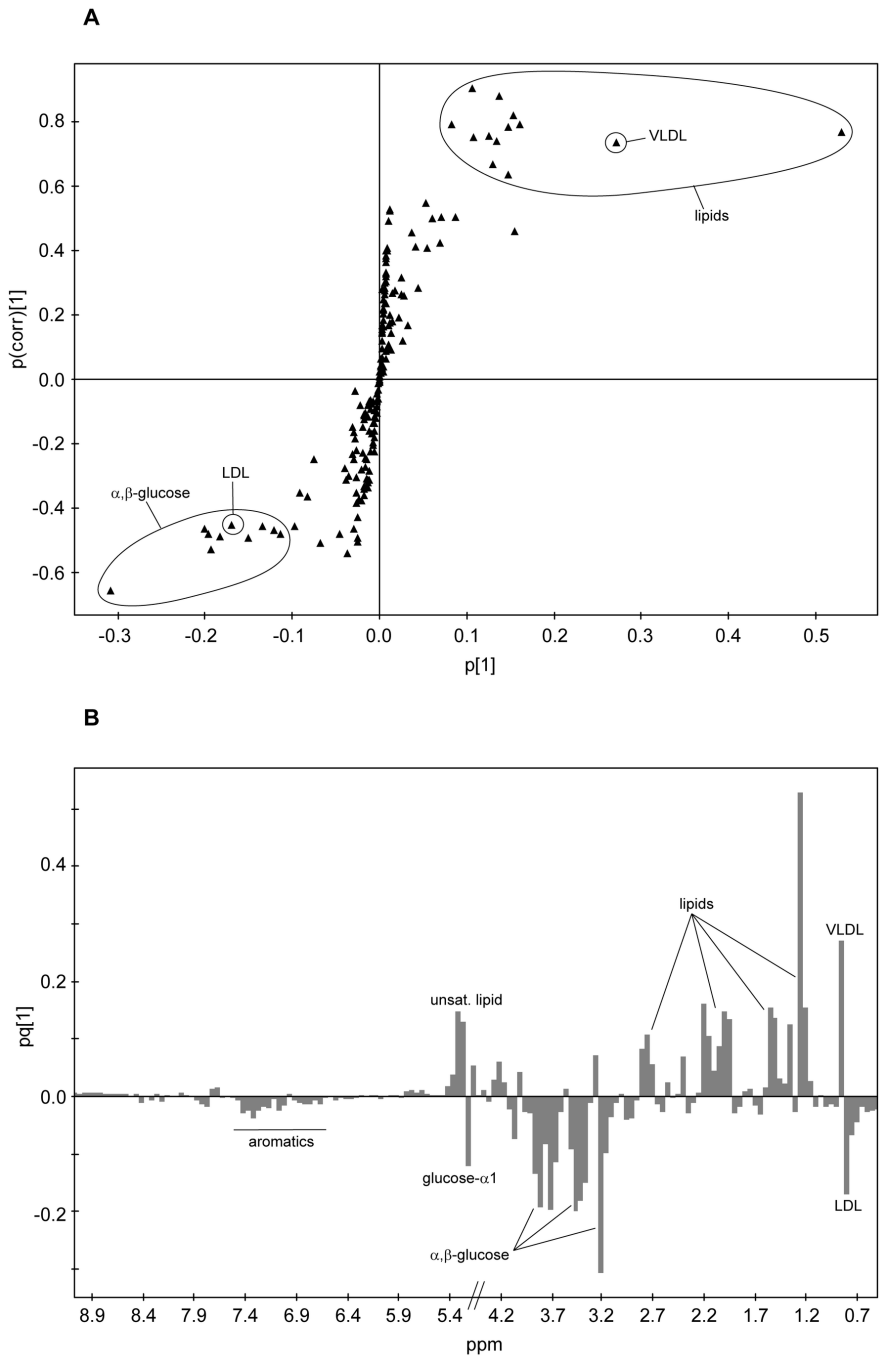
Early IUGR. (a) Representation of the OPLS-DA loadings in the form of an S-plot: the magnitude of each loading, p, is represented against its reliability p(corr).Those loadings (NMR buckets) showing high magnitude and reliability are encircled. (b) OPLS-DA loadings plot displayed as columns. Chemical shift is represented against magnitude of the loading. Reliability is evaluated by jack-knife 95% confidence intervals (not shown in the figure for clarity reasons). Positive loadings correspond with metabolites present in increased amounts in the case subjects; negative loadings correspond with metabolites in lower concentrations.

The metabolic information regarding the separation of case/control classes in the OPLS was extracted via SIMCA’s S-plot and loadings plot. The S-plot represents an S-shaped distribution of the NMR buckets (Figure 3a) that allows a quick visualisation of the OPLS discriminant analysis model for two classes. The axes from the predictive component, p[1] and p(corr)[1], represent the magnitude (modelled covariation) and reliability (modelled correlation) respectively. In spectroscopic data the magnitude of the signal is important since low magnitude peaks are close to the noise level and present a higher risk for spurious correlation. Potential good biomarkers would be represented by buckets showing simultaneously high effect and low uncertainty, that is, high reliability (very high or very low p(corr)[1]). Therefore, NMR buckets located at the tails of the S are the most attractive as they present high magnitude and high reliability. The S-plot corresponding to the early subset presents several variables (=NMR buckets) at both tails of the S. Those on the left correspond to metabolites that see their levels increased in the control samples (the controls are also located on the left side of the OPLS scores plot, Figure 2b), while the variables on the right correlate with metabolite levels increased in the early cases (located on the right side of the OPLS). 

**Figure 4:**
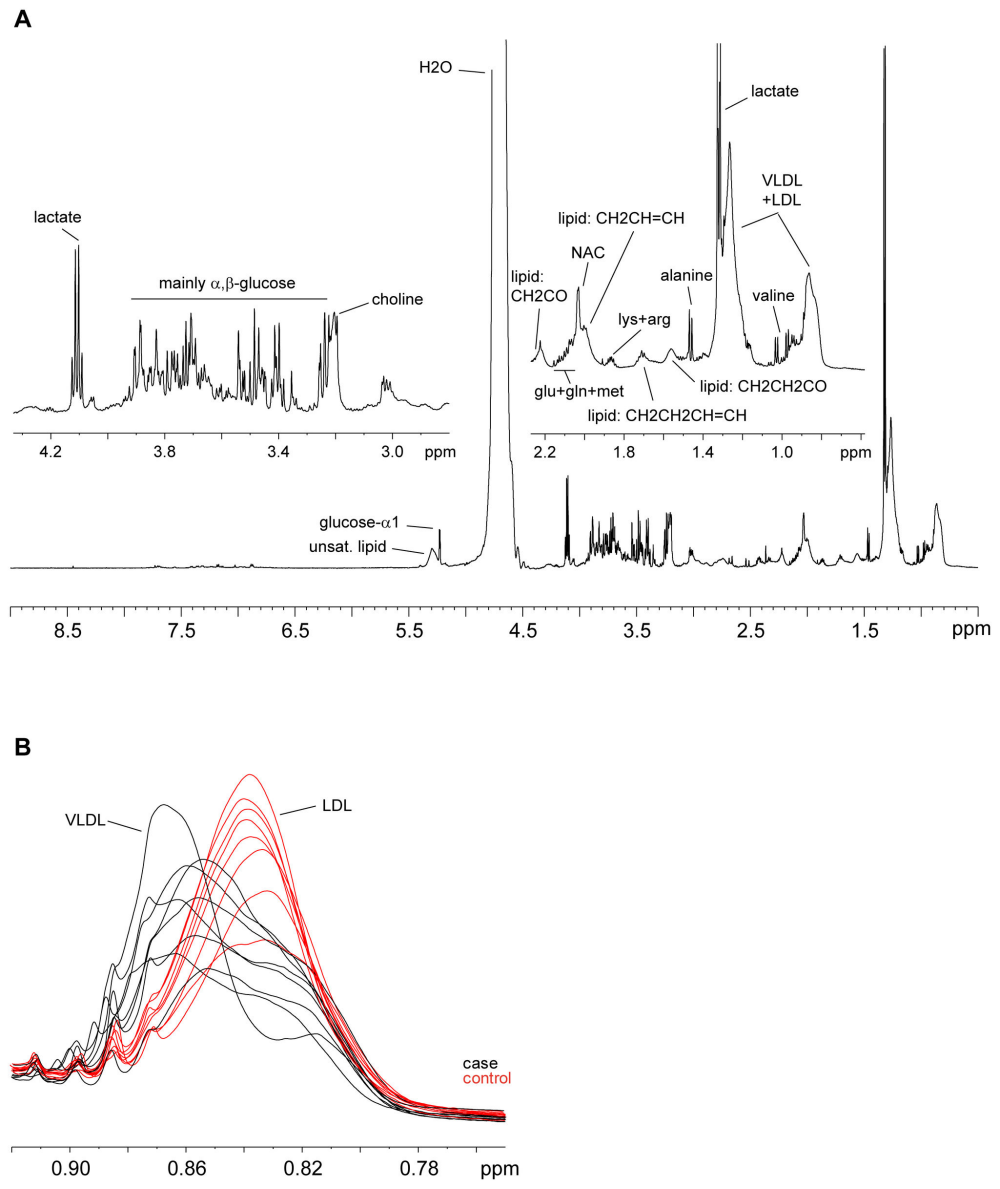
^1^H NMR of fetal serum. (a) Representative ^1^H-CPMG NMR spectrum of fetal serum from an IUGR early case at 600 MHz and 27°C. Insets: expansions of the aliphatic region (0.5-4.5 ppm) and assignment of the most abundant metabolites. LDL: low density lipoproteins; VLDL: very-low density lipoproteins; NAC: acetyl signals from glycoproteins. (b) Superimposed expansion of the VLDL/LDL CH_3_ region (0.90-0.78 ppm) from the ^1^H-CPMG NMR spectra, showing control (red lines) and case (black lines) samples from the early subset.

A representation of the effect that each bucket produces on the case/control separation is achieved in the OPLS-DA loadings plot depicted as a column plot (Figure 3b). The variables are ordered as in the original NMR spectrum and those columns presenting the largest positive/negative differences correspond to those metabolites contributing most to the class separation. In the early subset there are marked differences between cases and controls. Positive columns correlate with metabolites increased in the case subjects and negative ones correspond to metabolites increased in the controls. The reliability of each contribution is evaluated by jack-knifed confidence intervals (not shown in the figure). Those buckets that showed both large contribution and reliability in the S-plot/loadings plot were located in the NMR spectrum and analysed. The metabolites contributing to those buckets were identified via NMR through a combination of their ^1^H and ^13^C chemical shifts, ^1^H-^1^H coupling constants and spin system patterns obtained from 2D ^1^H,^13^C-HSQC, *J*-res and TOCSY experiments. The buckets presenting the largest increases when cases are compared with controls were correlated with various lipid molecules, whereas the highest metabolite reductions were observed for buckets related to α/β-glucose. In order to obtain a more precise evaluation of these variations, the NMR signals from metabolites identified as large contributors to the case/control separation were re-integrated accurately in the CPMG spectrum using variable-length bucket regions. Mean integrals + s.e.m. values were calculated separately for cases and controls, and their relative amounts compared (Table 6). The NMR signals corresponding to lipid molecules presented significantly higher levels (up to 75% increase) for the early IUGR cases compared to the levels shown by the controls. Among these variations the largest changes were observed for signals at 1.26 ppm (LDL+VLDL), 1.56 ppm (VLDL, triglycerides, CH_2_-CO), 2.20 ppm (triglycerides, CH_2_-CO) and 5.23-5.33 ppm (unsaturated lipids) (Figure 4a). Another signal that appears more intense in the IUGR subject samples (64% increase) is that corresponding with the methyl of ketone groups at 2.21 ppm (acetone/acetoacetate). More modest but statistically significant increases (aprox. 15%) are observed for creatine and glutamine. Regarding metabolite reductions, the levels of glucose are decreased (NMR signals at 5.22 and 3.90-3.40 ppm) for early IUGR cases compared to controls, with an average negative variation of 25%. Similar metabolite reductions are observed for choline and slightly lower for phenylalanine. A particular case was observed for lipid signals corresponding to VLDL and LDL in the region around 0.85 ppm (Figure 4b). This broad signal is contributed by both kinds of lipoprotein, but the resonance overlap does not allow a precise discrimination into two differentiated buckets. Although the % variation between cases and controls is very small when the whole region (0.90-0.79 ppm) is taken into account (4%), it can be easily discerned that the VLDL (left side) contribution is increased in IUGR early cases, whilst LDL levels (right) are higher in the control samples. NMR lipid signals are inherently broad and consequently partially suppressed in the CPMG experiment. However, any possible reduction in signal intensity caused by relaxation effects would be consistent across samples; thus, relative changes in concentration values would be still relevant. 

**Table 6:** Relative amounts of most significant metabolites in early-onset IUGR samples compared to control samples.

*metabolite*	*^1^H chemical shift (ppm)*	*assignment*	*mean+sem* (*cases*)**^*a*^**	*mean+sem* (*controls*)**^*a*^**	*p-value**^b^***	*% variation**^c^***
unsaturated lipids	5.35-5.24	=CH-CH_2_-CH	1.44E-02 ± 9.08E-04	9.83E-03 ± 3.82E-04	<0.0001	46.6
α/β-glucose	5.22. 3.89-3.40	H1-H6	2.41E-01 ± 1.70E-02	3.22E-01 ± 2.03E-02	0.0266	-25.1
lipid	2.82-2.70	C=CCH_2_C=C	9.60E-03 ± 4.25E-04	7.23E-03 ± 2.77E-04	0.0002	32.8
lipid	2.25-2.18	CH_2_CO	2.40E-02 ± 1.89E-03	1.37E-02 ± 4.84E-04	<0.0001	75.8
acetone/ acetoacetate**^*d*^**	2.21	CH_3_CO	2.23E-02 ± 2.45E-03	1.36E-02 ± 1.25E-03	0.0008	63.6
lipid (VLDL)	1.56	CH_2_CH_2_CO	1.35E-02 ± 1.05E-03	7.72E-03 ± 2.95E-04	<0.0001	75.0
lipid (VLDL+LDL)	1.30-1.20	CH_2_	1.17E-01 ± 9.03E-03	7.61E-02 ± 2.78E-03	<0.0001	53.6
lipid (VLDL+LDL)	0.88-0.78	CH_3_	6.46E-02 ± 3.03E-03	6.21E-02 ± 2.46E-03	NS	4.0
triglycerides	5.18	CHOCOR	3.97E-03 ± 2.40E-04	2.98E-03 ± 2.15E-04	0.0023	32.9
*myo*-inositol	3.28	H5	7.59E-03 ± 4.54E-04	6.67E-03 ± 3.18E-04	NS	13.8
choline	3.20	N(CH_3_)_3_	1.74E-02 ± 1.38E-03	2.34E-02 ± 7.25E-04	0.0001	-25.7
creatine	3.92	CH_2_	2.15E-02 ± 9.54E-04	1.85E-02 ± 5.79E-04	0.011	16.6
glutamine	2.44	γ-CH_2_	1.85E-02 ± 8.71E-04	1.62E-02 ± 5.52E-04	0.0141	14.8
phenylalanine	7.43-7.29	aromatics	2.34E-03 ± 6.57E-05	2.77E-03 ± 1.20E-04	0.0246	-15.3
tyrosine	6.88/7.18	aromatics	3.55E-03 ± 1.32E-04	3.82E-03 ± 2.00E-04	NS	-7.0
valine	1.03/0.97	CH_3_	4.69E-02 ± 2.05E-03	4.92E-02 ± 1.72E-03	NS	-4.7
leucine	0.95	CH_3_	3.12E-02 ± 9.05E-04	3.13E-02 ± 1.03E-03	NS	-0.3
alanine	1.47	CH_3_	1.16E-02 ± 6.32E-04	1.10E-02 ± 3.17E-04	NS	5.3

^a^ Integral values are normalised to the sum of total spectral intensity. ^b^ *p* values were calculated by Mann-Whitney *U* test. NS=Non-significant result (*p* value >0.05). ^c^ Variation of the relative amount of metabolite in subject cases relative to controls: positive value indicates an increase and negative value a decrease in the cases. ^d^ Overlapped with lipid signals. VLDL: Very low density lipoprotein. LDL: Low density lipoprotein.

Other metabolic differences have been reported in previous IUGR studies [25,26,37], involving molecules such as arginine, proline, glycine, serine, threonine, *myo*-inositol, sarcosine and creatinine. In our early IUGR data study, NMR buckets associated with these metabolites did not show any remarkable variation or statistically significant changes. 

### Late-onset IUGR vs. matched AGA newborns (late subset)

The same protocol described above was applied for the examination of the metabolic profile of the late subset comparing 56 late IUGR vs. 55 AGA controls. 

An initial unsupervised PCA of the whole late subset did not result in any obvious discrimination or clustering between cases and controls. Although several strong outliers were identified, only five samples were justifiably excluded from further analyses: a case sample with deficient water suppression in the NMR spectra that affected the base line of the whole spectrum and four controls that presented the same contamination found in some samples of the early subset. The PCA of the late subset excluding these five outliers (Figure S1) did not render any improvement in class separation or clustering of samples. The elimination of additional outliers from the analysis did not show any separation enhancement (PCA not shown). 

Analogously to the analysis carried out on the early subset, the PCA was also applied to the late samples for the examination of the maternal and neonatal clinical characteristics, but no clustering or group differentiation was observed. 

The subsequent supervised PLS/OPLS discriminant analysis on the late subset samples showed a uniform overlap between case/control classes (Figure S2a and Figure S2b), presenting very low goodness-of-fit and prediction values and poor external validation parameters (Table 5). A variable selection process was then applied by first removing from the analysis buckets from the NMR spectra that did not present any signal, and second by only retaining those variables that showed reliable (acceptable confidence-intervals) contributions. The resulting models did not improve the fitness and prediction values obtained with the full range of NMR variables, indicating that there was not a clear separation between cases and controls in the late subset. The metabolites identified in the early subset as relevant were also evaluated here; same metabolite variations as those found in the early subset were detected, but to a lesser extent (Table 7). Thus, an increase of lipid concentration was again observed for the IUGR cases, although in a lower variation (up to 20%) than in the early subset. Glucose levels were again slightly decreased (-5.8%) but the result could not be considered statistically significant. Other metabolites found relevant in the early subset study (acetone/acetoacetate, creatine, phenylalanine) did not present any significant difference in the late subset analysis. Although with relatively modest numbers, a particular case is presented by the amino acid glutamine which showed a 14.8% increase in the early IUGR cases, but a decrease of nearly 10% for the late IUGR subjects compared to the control samples. Similar reductions in metabolite levels were also observed for choline, alanine, valine, tyrosine and leucine. 

**Table 7:** Relative amounts of most significant metabolites in late-onset IUGR samples compared to control samples.

*metabolite*	*^1^H chemical shift (ppm)*	*assignment*	*mean+sem* (*cases*)**^*a*^**	*mean+sem (controls)**^a^***	*p-value**^b^***	*% variation**^c^***
unsaturated lipids	5.35-5.24	=CH-CH_2_-CH	1.41E-02 ± 4.76E-04	1.25E-02 ± 4.11E-04	0.0122	13.6
α/β-glucose	5.22. 3.89-3.40	H1-H6	2.50E-01 ± 1.12E-02	2.65E-01 ± 1.09E-02	NS	-5.8
lipid	2.82-2.70	C=CCH_2_C=C	9.68E-03 ± 2.57E-04	8.59E-03 ± 2.43E-04	0.0016	12.7
lipid	2.25-2.18	CH_2_CO	2.23E-02 ± 8.35E-04	1.87E-02 ± 6.45E-04	0.0002	19.3
acetone/ acetoacetate**^*d*^**	2.21	CH_3_CO	2.37E-02 ± 1.67E-03	1.99E-02 ± 1.05E-03	NS	19.6
lipid (VLDL)	1.56	CH_2_CH_2_CO	1.28E-02 ± 4.75E-04	1.10E-02 ± 3.51E-04	0.0017	16.5
lipid (VLDL+LDL)	1.30-1.20	CH_2_	1.14E-01 ± 5.00E-03	1.00E-01 ± 3.74E-03	0.027	13.8
lipid (VLDL+LDL)	0.88-0.78	CH_3_	6.60E-02 ± 1.90E-03	6.53E-02 ± 1.54E-03	NS	1.1
triglycerides	5.18	CHOCOR	3.76E-03 ± 1.47E-04	3.49E-03 ± 2.03E-04	NS	7.7
*myo*-inositol	3.28	H5	6.74E-03 ± 1.87E-04	6.58E-03 ± 2.17E-04	NS	2.3
choline	3.20	N(CH_3_)_3_	1.83E-02 ± 4.68E-04	2.09E-02 ± 5.48E-04	0.0003	-12.1
creatine	3.92	CH_2_	1.76E-02 ± 4.10E-04	1.78E-02 ± 3.46E-04	NS	-1.2
glutamine	2.44	γ-CH_2_	1.50E-02 ± 4.36E-04	1.66E-02 ± 3.83E-04	0.0124	-9.5
phenylalanine	7.43-7.29	aromatics	2.42E-03 ± 7.33E-05	2.43E-03 ± 2.02E-04	NS	-0.2
tyrosine	6.88/7.18	aromatics	3.22E-03 ± 1.07E-04	3.42E-03 ± 2.12E-04	0.007	-5.8
valine	1.03/0.97	CH_3_	4.37E-02 ± 1.13E-03	4.95E-02 ± 9.58E-04	0.0003	-11.8
leucine	0.95	CH_3_	3.20E-02 ± 5.74E-04	2.95E-02 ± 6.27E-04	0.0174	-7.9
alanine	1.47	CH_3_	1.05E-02 ± 2.77E-04	1.13E-02 ± 3.24E-04	0.035	-6.9

^a^ Integral values are normalised to the sum of total spectral intensity. ^b^ *p* values were calculated by Mann-Whitney *U* test. NS=Non-significant result (*p* value >0.05). ^c^ Variation of the amount of metabolite in subject cases relative to controls: positive value indicates an increase and negative value a decrease for the cases. ^d^ Overlapped with lipid signals. VLDL: Very low density lipoprotein. LDL: Low density lipoprotein.

**Figure 5:**
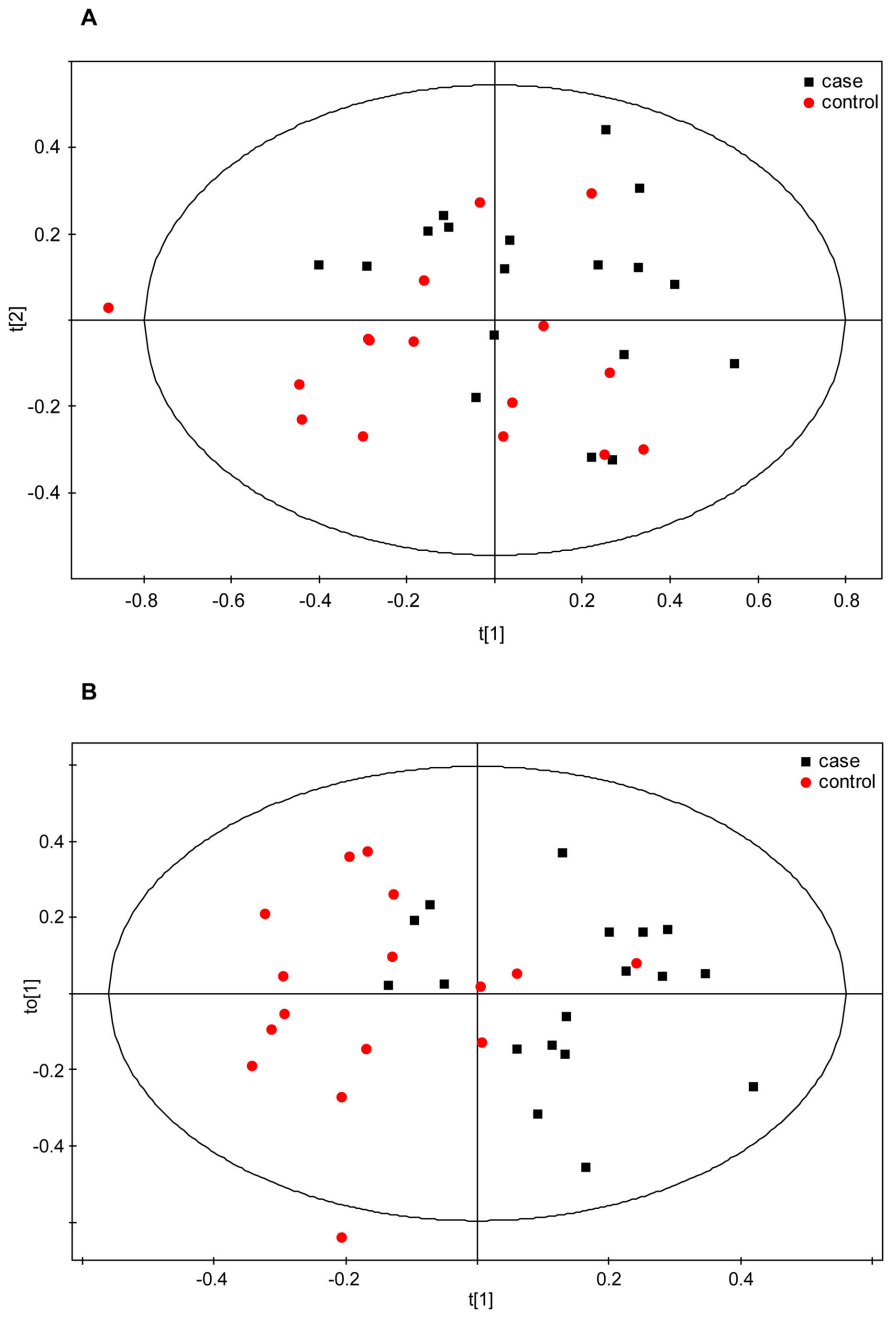
Multivariate statistical analysis of the late subset with cases showing increased brain vasodilation. (a) PCA scores plot of the ^1^H-CPMG spectra from controls (N = 17, red dots) and cases (N = 18, black squares). The first two principal components, PC1 and PC2, explain 43 and 20% of the variation, respectively. (b) OPLS-DA scores plot of the ^1^H-CPMG spectra from the same subset as (a). Samples from cases and controls were defined as two different classes. *R* ^2^ *Y* = 0.47; *Q* ^2^ = 0.32; *p*-value = 0.019.

**Figure 6:**
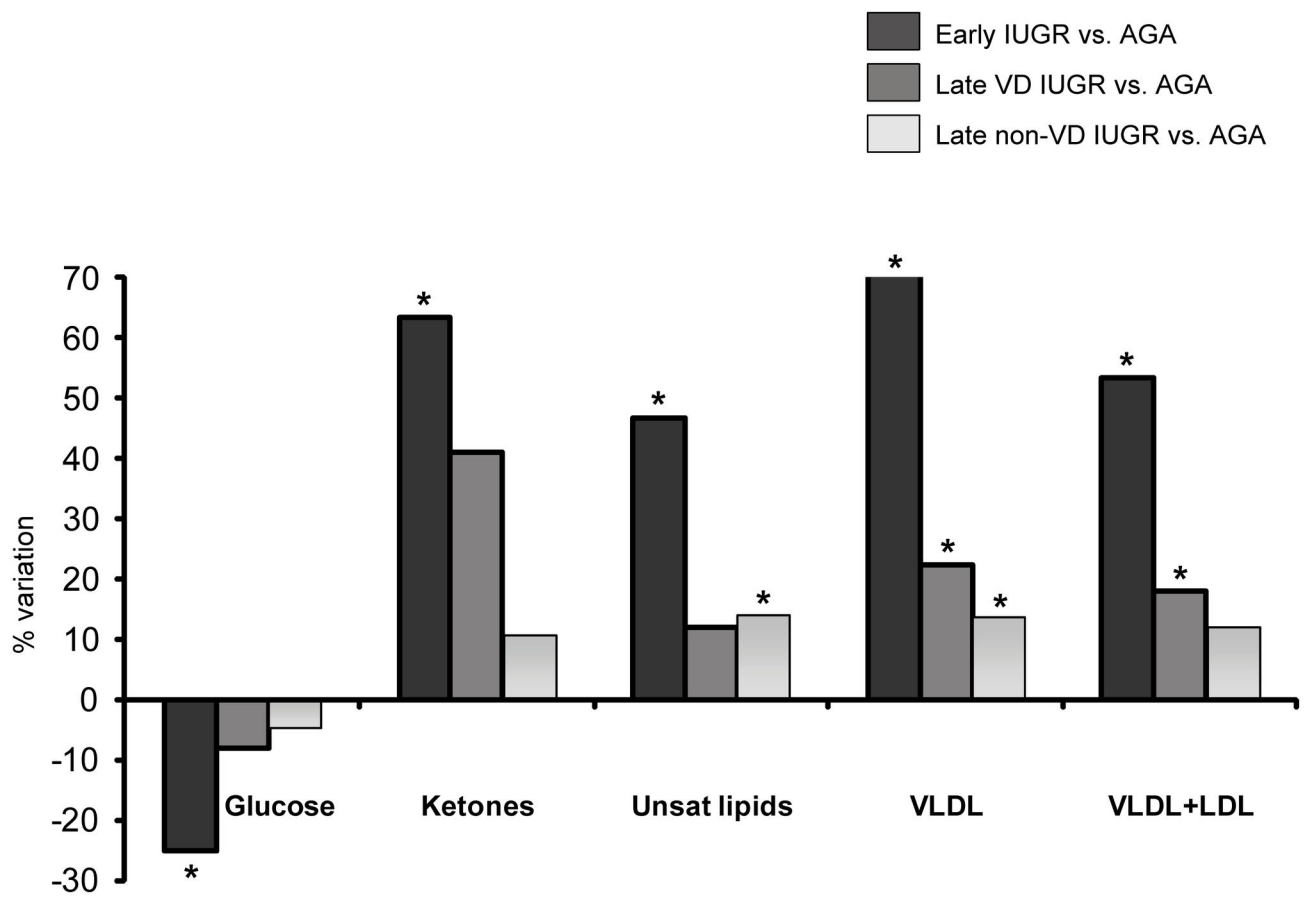
- Variation of the relative amount of lipids, glucose and acetone/acetoacetate (ketones) in early IUGR, vasodilated (VD) late IUGR and non-VD late IUGR cases relative to controls: positive value indicates an increase and negative value a decrease in the cases. * represents a statistically significant metabolic variation ( *p*< 0.05). Unsat.lipids= Unsaturated lipids. VLDL=Very low density lipoprotein. LDL=Low density lipoprotein.

**Figure 7:**
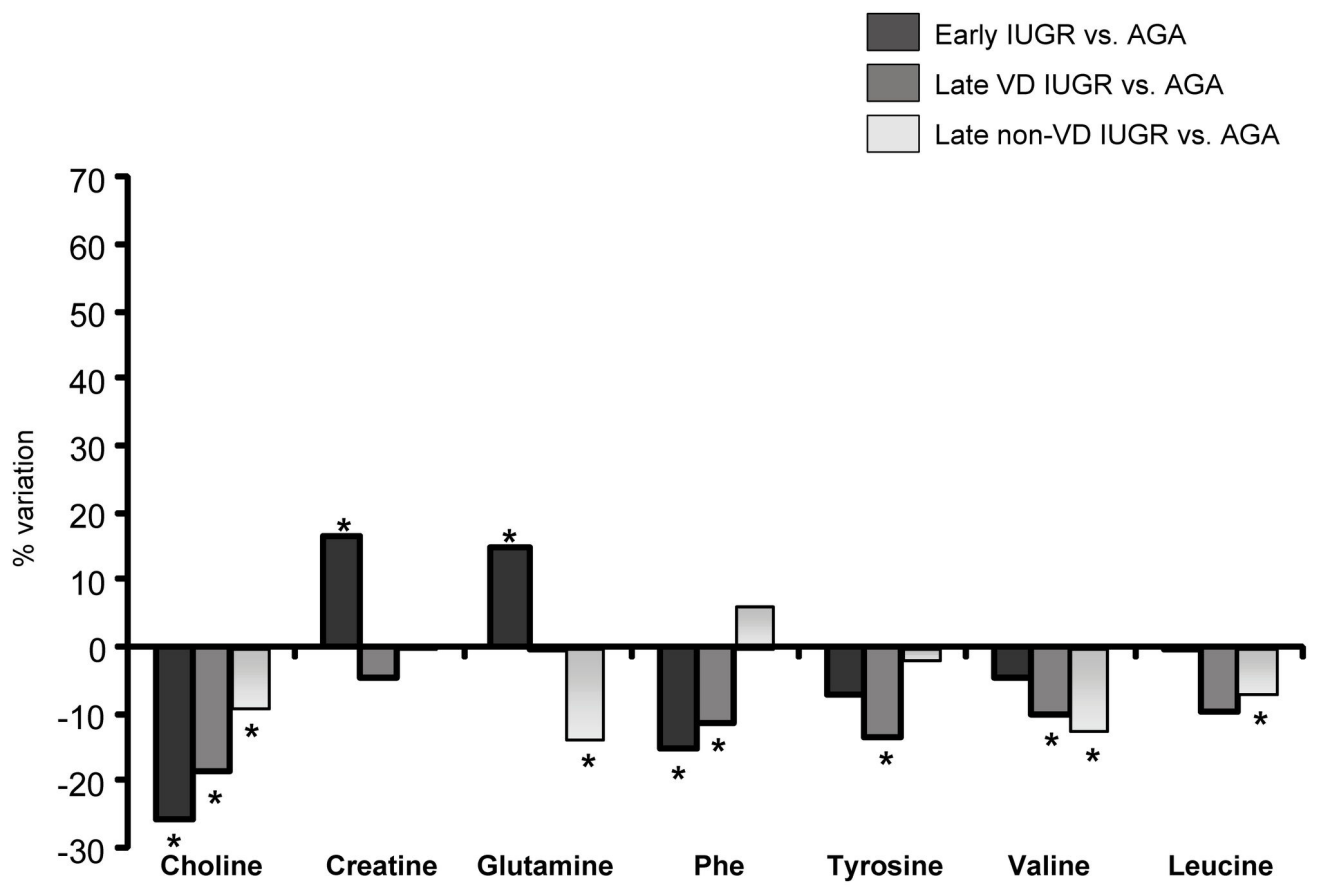
Variation of the relative amount of amino acids and derivates in early IUGR, vasodilated (VD) late IUGR and non-VD late IUGR cases relative to controls: positive value indicates an increase and negative value a decrease in the cases. * represents significant a statistically metabolic variation (*p*< 0.05).Phe =Phenylalanine.

Late samples were also divided in two subgroups based on the presence of brain vasodilation (Tables 3 and 4). 18 brain vasodilated late-onset IUGR cases matched with 17 AGA controls showed in the PCA some degree of separation between cases and controls (Figure 5a), with the larger portion of the cases located in the upper part of the score plot while the controls were found in the lower region. The multivariate supervised OPLS-DA confirmed these results and showed a discrimination between classes, although the model values could not be considered optimal (Figure 5b; Table 7: *R* ^2^Y = 0.47; *Q* ^2^ = 0.32). The metabolites causing the case/control separation were identified via the S- and loading plots obtained from the OPLS-DA model. Buckets with significant increases in the case samples were associated with lipids (Table 8; 2.25-2.18 ppm: CH_2_O; 1.56 ppm: CH_2_-CH_2_CO; 1.26 ppm: VLDL/LDL). In addition, decreases in the amounts of the amino acids choline, phenylalanine, tyrosine and valine were observed for the vasodilated late-onset IUGR cases relative to the control samples (Figures 6 and 7).

**Table 8:** Relative amounts of most significant metabolites in brain vasodilated late-onset IUGR compared to control samples.

*metabolite*	*^1^H chemical shift (ppm)*	*assignment*	*mean+sem* (*cases*)**^*a*^**	*mean+sem* (*controls*)**^*a*^**	*p-value**^b^***	*% variation**^c^***
unsaturated lipids	5.35-5.24	=CH-CH_2_-CH	1.35E-02 ± 5.33E-04	1.21E-02 ± 7.16E-04	NS	11.9
α/β-glucose	5.22. 3.89-3.40	H1-H6	2.54E-01 ± 1.81E-02	2.76E-01 ± 1.87E-02	NS	-8.1
lipid	2.82-2.70	C=CCH_2_C=C	9.32E-03 ± 2.82E-04	8.73E-03 ± 4.07E-04	NS	6.7
lipid	2.25-2.18	CH_2_CO	2.22E-02 ± 8.94E-04	1.79E-02 ± 8.10E-04	0.0006	24.0
acetone/ acetoacetate**^*d*^**	2.21	CH_3_CO	2.58E-02 ± 3.76E-03	1.83E-02 ± 1.46E-03	NS	41.0
lipid (VLDL)	1.56	CH_2_CH_2_CO	1.28E-02 ± 5.26E-04	1.04E-02 ± 4.58E-04	0.0031	22.5
lipid (VLDL+LDL)	1.30-1.20	CH_2_	1.10E-01 ± 5.23E-03	9.32E-02 ± 4.74E-03	0.0232	18.0
lipid (VLDL+LDL)	0.88-0.78	CH_3_	6.26E-02 ± 1.82E-03	6.42E-02 ± 1.82E-03	NS	-2.6
triglycerides	5.18	CHOCOR	3.57E-03 ± 2.17E-04	3.37E-03 ± 2.04E-04	NS	5.8
*myo*-inositol	3.28	H5	6.66E-03 ± 2.40E-04	6.86E-03 ± 3.45E-04	NS	-3.0
choline	3.20	N(CH_3_)_3_	1.72E-02 ± 7.28E-04	2.12E-02 ± 8.87E-04	0.007	-18.7
creatine	3.92	CH_2_	1.72E-02 ± 5.57E-04	1.80E-02 ± 6.94E-04	NS	-4.6
glutamine	2.44	γ-CH_2_	1.64E-02 ± 6.83E-04	1.65E-02 ± 7.32E-04	NS	-0.3
phenylalanine	7.43-7.29	aromatics	2.39E-03 ± 9.87E-05	2.70E-03 ± 8.97E-05	0.0174	-11.6
tyrosine	6.88/7.18	aromatics	6.32E-03 ± 2.98E-04	7.30E-03 ± 2.59E-04	0.0177	-13.4
valine	1.03/0.97	CH_3_	4.51E-02 ± 1.70E-03	5.03E-02 ± 1.80E-03	0.0367	-10.2
leucine	0.95	CH_3_	2.91E-02 ± 1.05E-03	3.23E-02 ± 1.15E-03	NS	-9.8
alanine	1.47	CH_3_	1.09E-02 ± 2.96E-04	1.07E-02 ± 2.29E-04	NS	2.1

^a^ Integral values are normalised to the sum of total spectral intensity. ^b^ *p* values were calculated by Mann-Whitney *U* test. NS=Non-significant result (*p* value >0.05). ^c^ Variation of the amount of metabolite in subject cases relative to controls: positive value indicates an increase and negative value a decrease for the cases. ^d^ Overlapped with lipid signals. VLDL: Very low density lipoprotein. LDL: Low density lipoprotein.

The multivariate analysis of the 38 non-vasodilated late-onset IUGR with 38 matched AGA did not produce a clear differentiation between cases and controls, presenting non-significant goodness-of-fit and prediction values. The comparison of metabolic levels rendered small positive variations for lipids/VLDL (Table 9, up to 17%) and minor reductions of the metabolites choline, glutamine, valine, leucine and alanine (Figures 6 and 7).

**Table 9:** Relative amounts of most significant metabolites in non-vasodilated late IUGR compared to control samples.

*metabolite*	*^1^H chemical shift (ppm)*	*assignment*	*mean+sem* (*cases*)**^*a*^**	*mean+sem* (*controls*)**^*a*^**	*p-value**^b^***	*% variation**^c^***
unsaturated lipids	5.35-5.24	=CH-CH_2_-CH	1.44E-02 ± 6.49E-04	1.26E-02 ± 5.05E-04	0.0164	14.3
α/β-glucose	5.22. 3.89-3.40	H1-H6	2.48E-01 ± 1.42E-02	2.60E-01 ± 1.35E-02	NS	-4.6
lipid	2.82-2.70	C=CCH_2_C=C	9.85E-03 ± 3.52E-04	8.53E-03 ± 3.05E-04	0.0031	15.5
lipid	2.25-2.18	CH_2_CO	2.23E-02 ± 1.15E-03	1.90E-02 ± 8.65E-04	0.0136	17.3
acetone/ acetoacetate**^*d*^**	2.21	CH_3_CO	2.28E-02 ± 1.73E-03	2.06E-02.± 1.37E-03	NS	10.8
lipid (VLDL)	1.56	CH_2_CH_2_CO	1.29E-02 ± 6.55E-04	1.13E-02 ± 4.65E-04	0.0286	13.9
lipid (VLDL+LDL)	1.30-1.20	CH_2_	1.16E-01 ± 6.91E-03	1.04E-01 ± 4.95E-03	NS	12.1
lipid (VLDL+LDL)	0.88-0.78	CH_3_	6.76E-02 ± 2.62E-03	6.58E-02 ± 2.09E-03	NS	2.8
triglycerides	5.18	CHOCOR	3.85E-03 ± 1.90E-04	3.54E-03 ± 2.83E-04	NS	8.5
*myo*-inositol	3.28	H5	6.77E-03 ± 2.52E-04	6.46E-03 ± 2.74E-04	NS	4.9
choline	3.20	N(CH_3_)_3_	1.89E-02 ± 5.83E-04	2.07E-02 ± 6.95E-04	0.0216	-9.0
creatine	3.92	CH_2_	1.77E-02 ± 5.43E-04	1.77E-02 ± 3.98E-04	NS	0.4
glutamine	2.44	γ-CH_2_	1.43E-02 ± 5.26E-04	1.66E-02 ± 4.54E-04	0.0009	-13.6
phenylalanine	7.43-7.29	aromatics	2.44E-03 ± 9.76E-05	2.30E-03 ± 2.90E-04	NS	6.0
tyrosine	6.88/7.18	aromatics	3.20E-03 ± 1.32E-04	3.26E-03 ± 2.97E-04	NS	-1.7
valine	1.03/0.97	CH_3_	4.30E-02 ± 1.45E-03	4.92E-02 ± 1.14E-03	0.0007	-12.6
leucine	0.95	CH_3_	4.30E-02 ± 1.45E-03	4.92E-02 ± 1.14E-03	0.0163	-7.1
alanine	1.47	CH_3_	1.04E-02 ± 3.79E-04	1.16E-02 ± 4.54E-04	0.0198	-10.6

^a^ Integral values are normalised to the sum of total spectral intensity. ^b^ *p* values were calculated by Mann-Whitney *U* test. NS=Non-significant result (*p* value >0.05). ^c^ Variation of the amount of metabolite in subject cases relative to controls: positive value indicates an increase and negative value a decrease for the cases. ^d^ Overlapped with lipid signals. VLDL: Very low density lipoprotein. LDL: Low density lipoprotein.

## Discussion

This study provides evidence on the existence of distinct metabolomic patterns of different clinical subsets in human IUGR. Using ^1^H NMR, several metabolites were identified allowing a clear discrimination between IUGR and AGA samples, consisting in important increases in lipids, acetoacetate and decreased glucose levels. Amino acid patterns showed significant changes in IUGR cases and among the study subgroups, supporting the existence of potential pathophysiological differences between early-onset and late-onset IUGR cases with and without signs of brain vasodilation.

One of the major findings in our work was the altered lipid profile. Abnormal lipid metabolism was detected in both early and late-onset IUGR samples, particularly at the expense of unsaturated lipids and VLDL. Our results are in agreement with previous reports of altered, pro-atherogenic lipid and cholesterol metabolism in IUGR fetuses [38–40]. These differences in the clinical setting have been tested in animal model studies obtaining similar results [41]. Furthermore, triglycerides (TGs) were found to be increased up to 33% in early-onset IUGR cases when compared to controls, which is consistent with previously published evidence [39,42–44]. The relevance of this finding relies on the fact that TGs act as an independent predictor of coronary artery disease which could, in association with the other lipid alterations found in this condition, potentially explain the proposed increased risk of cardiovascular disease in later life in IUGR cases [42]. As to the nature of the underlying pathophysiological mechanisms involved in these findings, abnormal lipid metabolism in IUGR could be explained by both chronic exposure to hypoxia and undernutrition: Hypoxic damage can induce significant derangements in plasmatic lipids during perinatal life resulting in hyperlipidemia [45] and also increased cholesterol esters in hypoxia-cultured placental tissue [46]. In addition, increased cholesterol levels are associated to starving conditions such as anorexia nervosa [47,48]. Moreover, reports from animal model in undernourished conditions show an increase of free fatty acids in arterial plasma and an impaired clearance of TG-rich lipoproteins [49]. All subgroups of IUGR were associated with significant changes in lipids, but there was a gradation of severity with more marked changes in severe cases (Figure 6). The presence of significant differences also in non-vasodilated late-onset IUGR cases supports the notion that at least a large proportion of these fetuses are not constitutionally small. 

In agreement with previous studies [41,50–53], early-onset IUGR showed significant decreases in glucose levels and significant increases in ketones. A non-significant trend was observed for late IUGR subgroups. Differences between early and late onset forms should be interpreted in line with previous findings in which glucose levels were inversely correlated to the clinical severity of IUGR [50,51,54]. As to the mechanisms involved in these findings, it is known that growth restricted fetuses maintain lower glucose concentrations to increase transplacental glucose gradient, which will keep the glucose uptake across the placenta of limited size [51]. Additionally, under growth restricted conditions glycolytic processes are enhanced by hypoxia-inducible factors [46], resulting in a more pronounced metabolic-substrate deficient situation with an increased ketogenesis. 

Specific changes in the amino acid profile provided an interesting insight which suggested important differences among the clinical subsets studied. Glutamine levels were found to be increased in early IUGRs as compared with matched controls. This finding is in line with previous studies in very low birth weight and also in early-IUGR preterm neonates [25,53]. Glutamine concentration in fetal blood depends on the transfer from maternal blood but also on placental synthesis [55]. Fetal glutamine is next to glucose as one of the main sources of cellular energy in fetal life [55]. It also plays a key role in fetal neurodevelopment being a precursor of alpha-amino butyric acid, a neurotransmission inhibitor [55]. Increased glutamine levels in severe IUGR could be explained by the inherent hypercatabolic status of this condition, in association to decreased glucose levels [56,57]. This lack of energy substrates may convey to increase glutamine supply to the fetus through different mechanisms [53]. When the different late-IUGR subsets were considered in regard to glutamine levels, thought-provoking differences were found: while vasodilated late-IUGR cases did not show any significant change, there was a significant decrease in the non-vasodilated late-IUGR cases (Figure 7). The explanation for this is unclear. However, this finding could be understood in the light of other pathologies with decreased glutamine levels such as in chronic stress conditions [58,59]. It is tempting to speculate that similar mechanisms may be involved here; whereas an overexposure to glucocorticoids is involved in chronic stress, it may as well play an important role in this clinical subform of IUGR. Indeed, increased corticosteroid transfer to the fetus has long been suggested as a potential pathogenic pathway of IUGR in humans [60,61]. 

Increased creatine levels in early IUGR could be interpreted as well by a metabolic-substrate deficient condition. Creatine is an essential metabolite for energy metabolism through the production of ATP and increased levels have been reported in preterm IUGR neonates [62] in IUGR-induced gilts [41].

Phenylalanine and tyrosine levels were decreased similarly in early and late vasodilated IUGR cases. Tyrosine is synthesized from the essential amino acid phenylalanine. Previously published results from in vitro and in vivo studies on phenylalanine in IUGR conditions have yielded controversial information [25,41,46,63], which could be explained by important differences in the selection of IUGR cases included. In general, studies focusing on early-onset severe IUGR cases showed decreased phenylalanine levels [63], attributed to an altered placental transport, plausibly resulting from a “damaged” placental tissue and the inherent hypercatabolic state in IUGR [41,63]. The observation of a similar decrease in phenylalanine in the group of vasodilated late-IUGR fetuses could support the hypothesis that this clinical subset represents a late form of placental insufficiency [16]. The implications of decreased phenylalanine and tyrosine as neurotransmitter precursors in these clinical forms, both associated with adverse neurological outcome [10,64–66], deserves further investigation. Finally, non-vasodilated late IUGR was associated with a remarkably distinct response in relation with phenylalanine and tyrosine suggesting a more prominent role of other mechanisms as discussed above.

Leucine and valine were significantly decreased in both forms of late IUGR regardless the presence or absence of brain vasodilation, but not in early IUGR. Valine and leucine, together with isoleucine, are defined as branched-chain amino acids (BCAA). The data goes in line with other reports from late IUGR fetuses in which all BCAA were decreased [67]. Our observations suggest that changes in these amino acids could be a characteristic feature of IUGR late in gestation. Again, diverse pathways might concur to explain these results. On one hand, plasmatic concentrations of BCAA are prominently reduced during chronic malnutrition [68,69]. However, exposure to increased glucocorticoids has similar effects to food restriction on BCAA aminotransferase and other enzymes regulating the catabolism and plasma concentrations of these amino acids [70]. The reason for the lack of changes in BCAA in early-onset IUGR is unclear. Previous studies suggest that BCAA could be transiently increased or remain unchanged during states of severe starvation [71], which might help explain our observations.

Decreased choline concentrations were found in all IUGR subsets, with a progression across clinical severity in early (25.7% decrease), vasodilated late (18.7%) and non-vasodilated late IUGR cases (9%). Choline is an essential metabolite under a high demand during pregnancy. Large amounts of this amino acid are delivered to the fetus across the placenta [72]. Circulating choline has also been reported to be reduced under malnutrition [73] and reductions in IUGR could be due to impaired transplacental supply. However, this does not exclude other pathways, since reduced serum levels have also been described in response to glucocorticoid therapy and chronic stress [74]. Choline has a key role in neurodevelopment, being the precursor of phosphatidylcholine for myelinization [75]. It has a crucial role in DNA methylation and hence in epigenetic mechanisms [72], which form the basis of fetal programming. Experimental [72,75] and human [76] studies suggest that choline-deficient conditions lead to abnormal cognitive development. Interestingly, all forms of IUGR, even those late-onset forms without signs of brain vasodilation, have been associated with long term neurodevelopmental and cardiovascular programming [77,78]. The potential role of choline deficiency in these long term effects deserves further investigation.

The main strengths of our study should be highlighted: Firstly, cohorts were constructed prospectively and cases were matched with controls by gestational age to avoid potential biases [40,50]. Also, clinical classification based on early and late IUGR is in agreement with our current practice and as shown in this study, the potentially different mechanisms involved in each clinical subform are worth this separation. 

However, we acknowledge that this study has several limitations. As expected, increased rates of preeclampsia and caesarean section delivery were present in the early IUGR cases. Regarding the first, this concurrence can be explained due to the known common ethiopathogenic mechanisms involved in preeclampsia and severe IUGR [43]; however, this clinical feature was not associated to any sample clustering or group differentiation based on the PCA that was performed to examine the influence of this variable. Furthermore, literature shows how the additional diagnosis of preeclampsia does not affect lipid alterations found in IUGR [39]. On the other hand, higher caesarean delivery rate in early IUGR can be explained due to a usually undeferrable need to end the pregnancy at a distant gestational age from term with unfavourable conditions for a vaginal delivery. Natural control patients for this condition are those born prematurely due to preterm labour, which favours vaginal delivery. Therefore, early IUGR is inevitably associated to a high rate of cesarean delivery. However it should be noted that samples from the late subset, with similar caesarean section rates between cases and controls, showed similar but less-severe metabolic differences than the early subset, suggesting that metabolic differences may be related to the disease itself and not to the mode of delivery. So, we acknowledge that residual metabolic effects from the additional diagnoses of preeclampsia or caesarean delivery to IUGR can not be completely ruled out. Also, we acknowledge that all clinical classifications are subject to bias and an important overlapping among cases is inevitably present. In addition, we used here three subsets of IUGR but future research might identify further subgroups either among early or late-onset forms of IUGR. For instance, it is likely that among late-onset there are constitutionally small fetuses which are not the result of any pathological condition. We expect that future studies will extend our observations to improve the characterization of pathogenic pathways leading to the common problem of restricted intrauterine growth. 

To conclude, IUGR is not associated with a unique metabolic profile, but important changes are present in different clinical subsets used in research and clinical practice. Besides major and common changes found in most IUGR cases, such as increased lipids and decreased glucose, amino acid changes may bring insight to specific mechanisms involved in this disease. It seems that early- and late vasodilated IUGR share features with respect to some amino acid changes that are not observed in late non-vasodilated fetuses. These results provide support to the hypothesis that early-onset and late-onset vasodilated fetuses share a similar pathophysiology with differences in the timing and severity of the disease [16,19,78,79]. However, at the same time there are common features to both late-IUGR subsets, which are not observed in early-onset IUGR fetuses. Further research is required to identify the origin of such changes in late-onset IUGR, since as discussed above, diverse pathways could lead to similar effects. These results provide grounds for future studies aiming at characterizing comprehensively specific hormonal and metabolic alterations underlying different pathophysiological subsets within the common diagnostic label of intrauterine growth restriction. Such knowledge is required to advance in the development of biomarkers and identify therapeutic approaches.

## Supporting Information

Figure S1 **Multivariate statistical analysis of the late subset.** PCA scores plot of the ^1^H-CPMG spectra from controls (N = 51, red dots) and cases (N = 55, black squares). The first two principal components, PC1 and PC2 explain 36 and 30% of the variation, respectively.
(TIF)

Figure S2 **Multivariate statistical analysis of the late subset.** (a) PLS-DA (*R* ^*2*^ *Y* = 0.22; *Q* ^2^ = 0.07; *p*-value = 0.133, statistically non-significant) and (b) OPLS-DA (*R* ^*2*^ *Y* = 0.30; *Q* ^2^ =0.17; *p*-value = 0.005) scores plots of the ^1^H-CPMG spectra from the controls (N = 51, red dots) and cases (N = 55, black squares). Samples from cases and controls were defined as two different classes.
(TIF)

Table S1 **Maternal drug administration in early IUGR subset.**
(DOC)

Table S2 **Maternal drug administration in late IUGR subset.**
(DOC)
