# Supplementary figures and images for: Metabolomic Profile of Umbilical Cord Blood Plasma from Early and Late Intrauterine Growth Restricted (IUGR) Neonates with and without Signs of Brain Vasodilation
Source: PLoS One. 2013 Dec 2;8(12):e80121. doi: 10.1371/journal.pone.0080121 (PMC3846503; doi:10.1371/journal.pone.0080121)

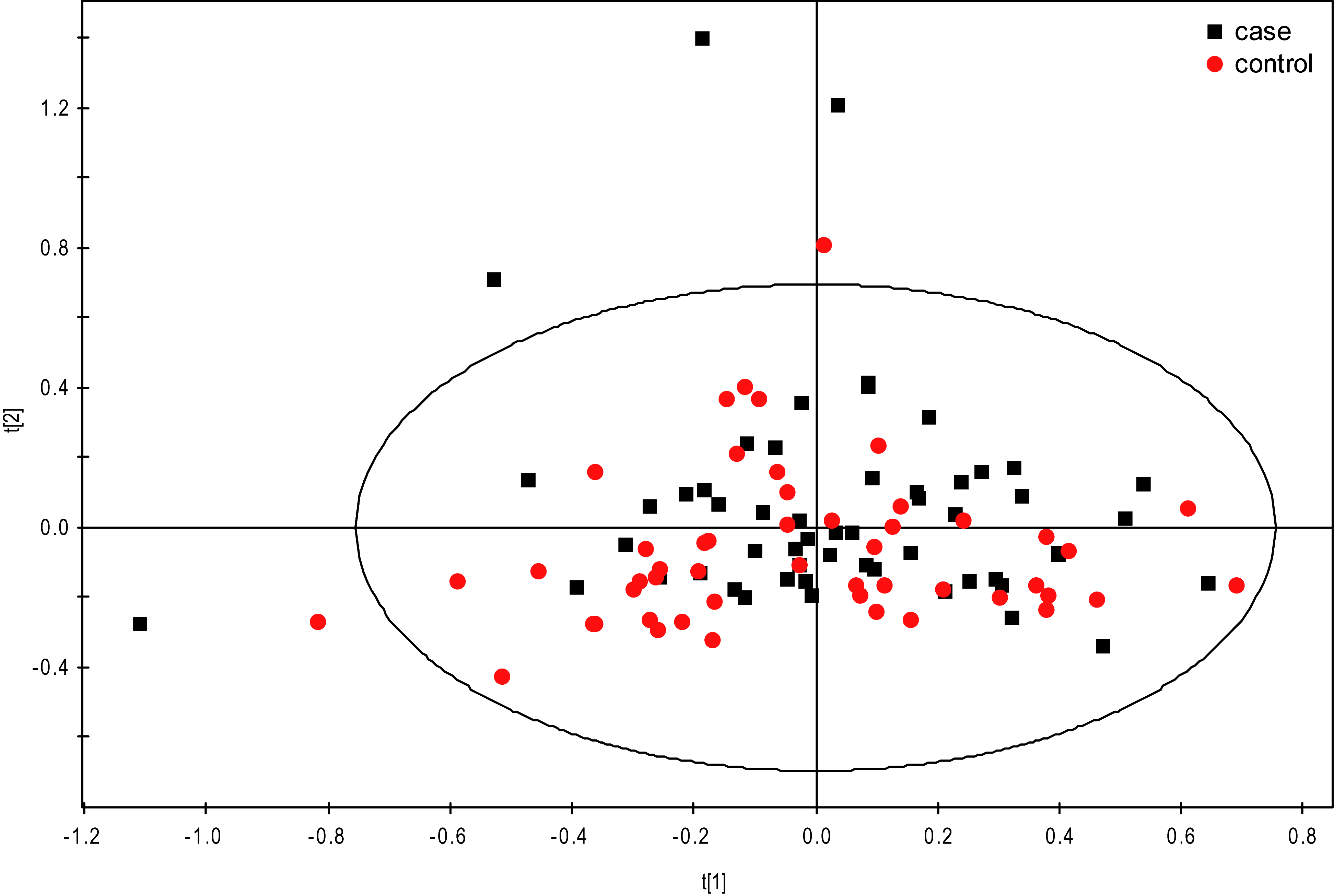

Supplement: Figure S1 — Multivariate statistical analysis of the late subset. PCA scores plot of the 1H-CPMG spectra from controls (N = 51, red dots) and cases (N = 55, black squares). The first two principal components, PC1 and PC2 explain 36 and 30% of the variation, respectively. (TIF) [file pone.0080121.s001.tif]

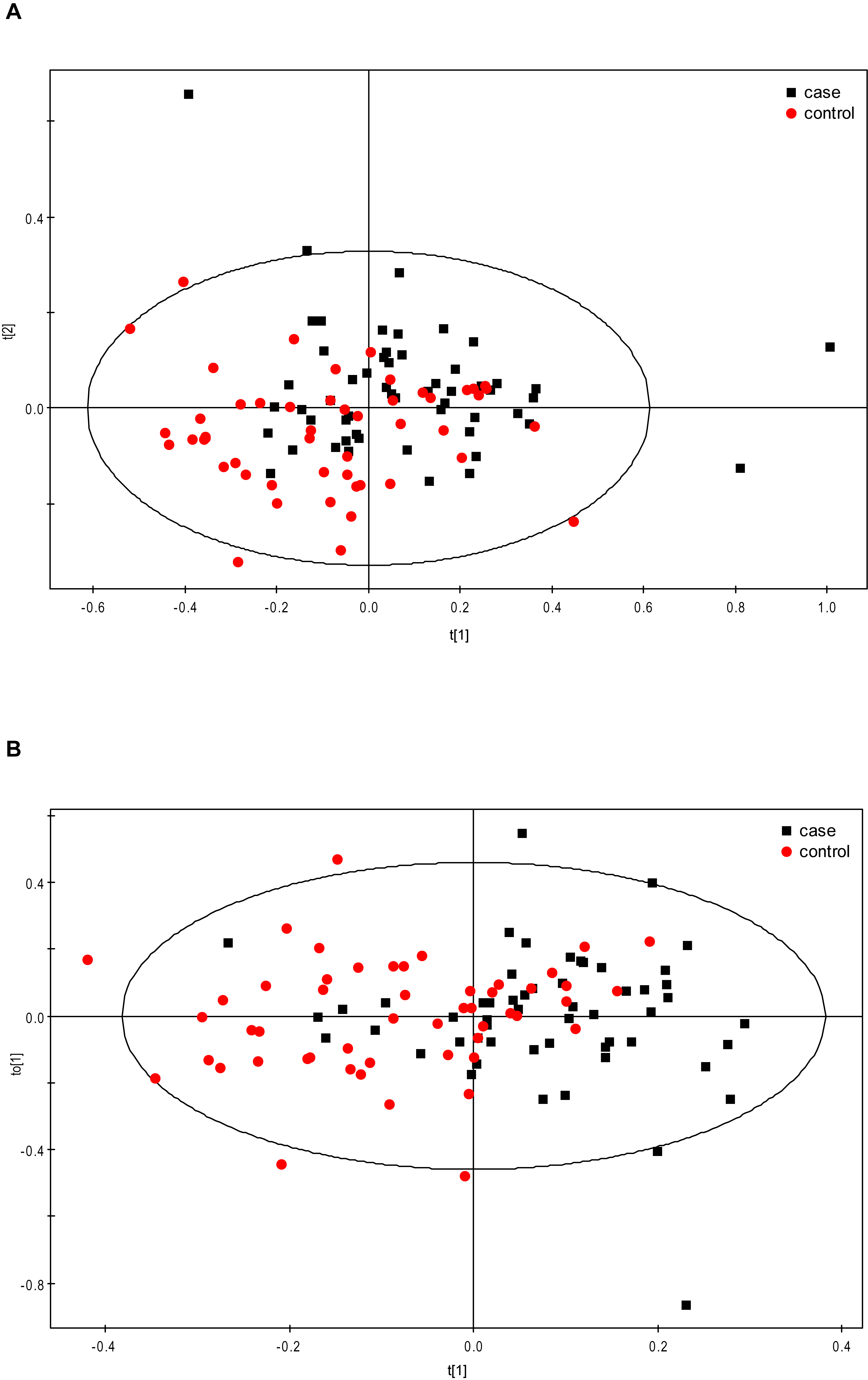

Supplement: Figure S2 — Multivariate statistical analysis of the late subset. (a) PLS-DA (R 2 Y = 0.22; Q 2 = 0.07; p-value = 0.133, statistically non-significant) and (b) OPLS-DA (R 2 Y = 0.30; Q 2 =0.17; p-value = 0.005) scores plots of the 1H-CPMG spectra from the controls (N = 51, red dots) and cases (N = 55, black squares). Samples from cases and controls were defined as two different classes. (TIF) [file pone.0080121.s002.tif]
