# Supplementary material for: Metabolomic Profile of Umbilical Cord Blood Plasma from Early and Late Intrauterine Growth Restricted (IUGR) Neonates with and without Signs of Brain Vasodilation
Source: PLoS One. 2013 Dec 2;8(12):e80121. doi: 10.1371/journal.pone.0080121 (PMC3846503; doi:10.1371/journal.pone.0080121)
Supplement: Table S1 — Maternal drug administration in early IUGR subset. (DOC) [file pone.0080121.s003.doc]

Table S1- Maternal drug administration in the early subset expressed as proportion of patients that received specific medication.

|  | **Early IUGR**  **(N=20)** | **AGA**  **(N=23)** | ***P*** |
| --- | --- | --- | --- |
| **MgSO_4_** | 40 | 0 | <.01 |
| **Antibiotics**  (amoxicillin-clavulanic acid, penicillin, erythromycin, ampicillin or gentamicin) | 10 | 73.9 | <.001 |
| **Tocolysis**  (ritodrine, nifedipine or atosiban) | 15 | 60.9 | <.01 |

Results are expressed as percentages determined by Pearson’s *X*^2^ or Fisher’s exact test as appropriate. MgSO_4_: Magnesium sulphate.
